# Jonathan Yewdell Discusses Viral Immunology, Vaccine Development, Navigating a Scientific Career, and Offers Perspectives on Transforming Scientific Publishing and Research Education

**DOI:** 10.20411/pai.v9i2.753

**Published:** 2024-09-27

**Authors:** Neil S. Greenspan

**Affiliations:** 1 Case Western Reserve University, Cleveland, Ohio

**Keywords:** Immunology, influenza A, viral immunology, antibodies, postdoc training

## Abstract

In this interview, Jonathan Yewdell talks with *Pathogens and Immunity* senior editor Neil Green-span about the evolution of viral immunology, highlighting his work and the contributions of other influential scientists. He emphasizes the importance of passion and collaboration in scientific research, illustrating the potential for groundbreaking discoveries through networking. He provides advice on navigating a scientific career, stressing the significance of strong mentorship. And he shares his perspective on transforming the scientific publishing industry and research education.

## NEIL S. GREENSPAN, MD, PHD

I am Neil Greenspan on behalf of *Pathogens and Immunity*, for which I am one of the senior editors. Today, it is my pleasure to host a conversation with Jonathan Wilson Yewdell, MD, PhD, Chief of the Cellular Biology Section of the Laboratory of Viral Diseases, which resides within the National Institute of Allergy and Infectious Diseases. By way of full disclosure, I’ve known Jon for many years, since the fall of 1975, as we were classmates and, for 3 years, roommates while attending both medical and graduate school.

Jon received his undergraduate degree in biochemistry magna cum laude from Princeton in 1975, and he completed his M.D. and Ph.D. degrees at Penn in 1981. Jon is a highly accomplished investigator straddling the areas of immunology, virology, and cell biology as they relate primarily to viral infections, with a special focus on influenza A viruses. He has made major contributions to characterizing and understanding aspects of both humoral and cell-mediated immunity to influenza A viruses, as will become evident in the discussion to follow. I am delighted to have this opportunity to learn more about the insights Jon has generated from his decades of experimental inquiry. Jon, welcome to this conversation for *Pathogens and Immunity*, where Jon is an associate editor.

To get started, what early influences steered you to science in general and to the intersection of immunology, virology, and cell biology in particular?

## JONATHAN W. YEWDELL, MD, PHD

Good question. A series of random events. As a biochemistry major at Princeton, I wrote 3 required junior papers: each professor assigned an original scientific publication for dissection. One, I wrote for Arnie Levine. He also taught an amazing graduate-level course in virology, probably one of the first college courses in virology. There weren’t a lot of graduate students at Princeton at that time, so it was mostly undergraduates. I wound up loving virology and joining Arnie’s lab for my senior thesis, also a Princeton requirement. At that time, Arnie was working on adenovirus and SV40 virus (simian vacuolating virus 40). He would later become famous as one of the scientists who discovered p53, which is the most studied protein now, I think. Back then, he was an associate professor and getting his career going with a great lab. Arnie was interested in how hamsters could reject adenovirus-transformed tumors — and, as you know, he wound up studying that for the next 50 years.

If I had ended up in someone else’s lab, I’d be having a conversation with someone else at this point. Life is like that. There are these decision points, and you’re not aware of which ones are important until you look back.

Because Arnie was my mentor, I got into the University of Pennsylvania (Penn) Med School. He had completed his PhD at Penn, and he wrote a strong letter for me. When I was accepted, I asked Arnie who I should work with. His best friend, I think from graduate school, was Norman Klinman. That’s how I wound up in Norman’s lab.

Arnie knew Penn quite well and thought immunology was the strongest department, so that directed me into immunology, and then virology and cell biology came later.

## NSG

Do you recall any particularly influential events in your undergraduate or graduate training?

## MAJOR INFLUENCES AND LESSONS LEARNED DURING TRAINING

### JWY

In Arnie’s lab there was this somewhat peculiar graduate student, Art Levinson, who would become *the* Art Levinson, legendary CEO at Genentech [1]. You never would have guessed this if you knew him as a graduate student. People are filled with all kinds of potential that is not obvious.

I just saw Arnie for the first time in about 40 years, and we were talking about Art. I didn’t really know that much of Art’s career at Genentech. He was an interim president, because the president had been canned. They didn’t think Art would be a great president, even though he was one of their first employees. You only really saw how good he was once he became the head.

### NSG

Well, it sort of goes to your point about random events, because if that other President hadn’t been canned, he might never have been elevated.

### JWY

Art took me under his wing, and we talked science all the time. I think he had a big part in me falling in love with science as an undergraduate.

The second influential event was in med school. After a few weeks, I realized I kind of hated medicine. That’s a problem if you’re a medical student. And, unlike you, I wasn’t in the MD-PhD program. From the age of 12, I had this immature dream that I wanted to be a primary care physician, of all things. And you, more than anyone, know how ill-suited I am for that. Sometimes it takes a while to learn these things about yourself.

After starting medical school, it took a few weeks to recognize my mistake. I wasn’t going to drop out. Penn, in their wisdom, held a couple of MD PhD slots open for people who were already in medical school, which I think was a brilliant idea. That’s how I became an MD, PhD. And then, as I said, Arnie steered me to Norman’s lab. Norman was a fantastic immunologist. He wasn’t the best mentor, but it was a great lab, he was doing brilliant work. And he doesn’t get enough credit, I think, for being the first one, really, to work with monoclonal antibodies [2].

### NSG

It’s interesting that he was phenomenal intellectually and, in the lab, as far as I know or could recall, and he just didn’t have visibility the way some people do.

### JWY

He lost it. I think back then he was a rising star. When he moved to Scripps, which was the second random event that shaped my career, he lost visibility. Before there were hybridomas, there was his splenic focus system, first system where you could get monoclonal antibodies in solution. There was Jerne’s plaque assay, which Gus Nossal was doing as well. But those antibodies were secreted by B cells into agarose, and you couldn’t study them biochemically.

### NSG

You’d be able to see what the antibody could do, but you didn’t have a supply of it.

### JWY

Right, and Norman must have had amazing hands, because he was purifying these tiny amounts of antibody and labeling them with I^125^. He was doing immunochemistry. I think he was the first one to show the homogeneity of monoclonals in the Sips plot versus the heterogeneity of polyclonal sera [3].

### NSG

Yeah, because most people wouldn’t have had a monoclonal antibody (of known specificity) to do that.

### JWY

Norman trained with Fred Karush, who got a couple of things wrong, like, the selective antibody theory! But Fred was a great scientist as well.

### NSG

Well, as I recall, Fred was as responsible as anyone for really putting immunochemistry, as it was then known, on a firm thermodynamic footing.

### JWY

Right and monogamous bivalency, which is a key concept in immunology [4].

### NSG

It’s interesting because people don’t use Eisen’s textbook anymore [5]. Most of them don’t know very much about how antibodies work.

### JWY

The funny thing is that, as textbooks expand with new knowledge, you would think they would always incorporate what was there before, but they don’t.

### NSG

The biophysical side of immunology is not very well represented in most textbooks. They’re still very high quality in other respects.

### JWY

You can still buy a copy of “Microbiology” by Davis, Dulbecco, Eisen, and Ginsburg with Eisen’s portion. On Amazon, you can get it for $12.54 [6].

### NSG

That was probably the best introduction to how to think about antibodies.

### JWY

Eisen was brilliant. In any event, I don’t know if you remember this, but Norman got divorced, and his wife moved to UC Berkeley. My understanding was that to be closer to his kids he moved to Scripps in La Jolla. I asked him what I should do, and he said, “Why don’t you go talk to Walter Gerhard?” That’s how I wound up in Walter’s lab.

In Norman’s lab, I was doing something with *Streptococcus* with antibodies. I didn’t particularly dislike bacteria, but I really love viruses. Moving to Walter’s lab was lucky because, even though I had been doing straight immunology with Norman, I became the house virologist in Walter’s lab. That was an incredibly fortunate, good match. Walter was not a perfect human being, as you know, but he was an outstanding mentor in many ways

### NSG

It’s interesting, too, because at that stage, virologists were ascending in importance in biomedical science. And bacteriologists were descending. That was before the boom in molecular pathogenesis (of bacteria).

### JWY

Well, that’s when hubris was dominating medicine, and the MDs thought they had conquered infectious diseases. I think the interest in viruses was driven more from using them as tools for expressing genes and figuring out how they worked at molecular and cellular levels. Additionally, there was a strong belief, among some anyway, that viruses were a major cause of cancer, which took decades to come to fruition. Molecular genetics was pretty much started by virologists. I think that was all a part of it. Arnie was in that club — the guys using viruses to study molecular genetics. And then what really put virology back on the map since the turn of the 19th to 20th century, was HIV. Smallpox was eradicated in 1978. And people thought, “Okay, well, we don’t have to worry too much about viruses at this point.” And then HIV comes back and, it wasn’t obvious at first, but within about 5 years, it became clear that it was the single most pathogenic virus that humans have faced. If you had the CCR5 co-receptor, there was almost a 100% mortality rate before there was treatment.

In the 70’s, The Wistar Institute was the place for viral immunology. Hilary Koprowski was the head of the institute where you and I were doing our PhDs. Hilary was a very interesting character. He had a lot of talents, and among them, was picking out good scientists. Walter was one of them.

### NSG

It’s actually very interesting to reflect on that because, going back to visibility, Walter was way less visible than Norman. He was a phenomenal investigator, but there’s no graduate student I know who knows who Walter Gerhard is. He was crucial for the unraveling of some of the (fundamentals of) viral immunology.

### JWY

Absolutely. Not only was he the first one to make hybridomas against viruses, he was the first one to work with them, and he did some of the best experiments. That work isn’t remembered terribly well, but it hasn’t been surpassed. In the end, one of the major contributions of scientists to science is the people they train. Who today knows who Stephen Fazekas de St. Groth was? He’s as smart a biologist as anyone whose papers I’ve ever read. He was the one who trained Walter (and Rob Webster), and I am a direct descendant of that way of thinking. The project Walter put me on was straight out of Fazekas’ brain. The lineage lives on regardless of who remembers it.

### NSG

Have they continued mutating?

### JWY

Yes, thankfully. Absolutely. You know, the family tree — some of them do go extinct, but the family tree extends considerably.

There were other great viral immunologists at Wistar: your mentor Peter Doherty of course, Barbara Knowles, Giorgio Trinchieri, who just was elected to the USA National Academy, and other amazing scientists. At that time, there wasn’t a lot of viral immunology in the world, and Wistar was one of the few centers. You had the John Curtin School of Medical Research in Australia, where Peter had come from, Wistar, Mill Hill in London.

### NSG

Interestingly, it incorporated, as you have done over your career, both the antibody side and the T cell side. It was really Peter and Rolf Zinkernagel who made the T cell side worth pursuing.

### JWY

Well, everything was just being discovered. That was the joke—my undergraduate thesis was never going to work because it was T cells that were doing the job in getting rid of adenovirus transformed cells, not antibodies as we believed.

### NSG

You were looking at antibody?

### JWY

Yes, collaborating with Norman. He was sending us a rabbit anti-mouse antibody conjugate. So, you know, once every two weeks or so, someone had to drive it up from Norman’s lab in Philly to Princeton, because it was radioactive.

### NSG

That’s interesting because it shows how what looks like an incredibly reasonable project at a given stage in the development of an area of inquiry can turn out to be just totally misguided.

### JWY

My undergraduate project was useless, except for training. For training, nothing matters. All that matters for training is to design and interpret experiments. It’s ironic that when you’re a student, it’s better when things don’t work. You must figure out how to make it work. When things work too well, you don’t learn as much. And ideally, there’s a balance between things working and not working. You need some success, to provide, if nothing else, positive feedback to keep going. But the main thing of training is to be trained and not necessarily to produce papers.

### NSG

Years ago, I was on a study section that reviewed postdoc fellowship F32 proposals—I think that’s what they were called. And there was a real emphasis not just on the quality of the proposed science, but also on the training value. And that was a huge part of it. It was not taken lightly. It was a real central focus of the study section that there be training value in the project being proposed.

That was quite a fun discussion. Let’s move on to the next question. Looking back, do you think your medical training, even though you weren’t thrilled, provided any benefits in terms of your research career?

### JWY

So, people may not appreciate how bad a medical student I was, but you do. You know, the old joke: what do you call the person who is last in their med school class? Doctor! I proved that it’s almost impossible to flunk out of medical school, unless you are truly psychotic. Stan Plotkin, as you might recall, signed my evaluation in pediatrics that stated that I needed psychiatric help. Before doing that — I still hold it against him, that really could screw up a career—he should have talked to me, and maybe he would have thought, “he’s even crazier than I thought”. In any event, that was one of the last of the med school clinical rotations where I routinely showed up. Not that there were that many courses, because as you know, at Penn, you could graduate, I think, with just 13 months of clinical rotations.

### NSG

Although I wasn’t quite as negative about it, as you, I was pretty interested in the research track. And I think I graduated with my MD with about the minimum number of clinical rotations.

### JWY

Yeah, it wasn’t much but, even with that minimal requirement, I spent most of my time in the lab when I was supposed to be in the clinic. But still, there were huge benefits from finishing med school. You get an emotional education in medical school, which I don’t think you can match in any other venue. First, the intense relationship with the other students. Penn was a big medical school; there were still only 164 of us to start. You make so many fantastic friends there; you might meet your wife there, which I did. And then you must deal with doctors, who I didn’t like; patients, who I loved; and the nursing staff, who I really liked. You interacted with all these different kinds of people.

And the part of medicine I liked most, taking medical histories from patients. I didn’t like scut work assigned by interns and residents. My opinion was that I was there to learn and not to do menial labor. So, I was a disaster for the residents who depended on the medical students to do things that the hospital should have done for them. At Penn, even though it’s a great medical school, certainly one of the more intellectual medical schools in the world, a substantial fraction of the faculty had an anti-intellectual attitude. You’d ask an insightful question. If you’re smart, you know what a good question is. Something students don’t often realize at that point, they’re smarter on average than those higher in the hierarchy. Because after age 21 or so, your cognitive function starts to decline. It’s slow at first and then you hit 50 or 60 and the decline is rapid. So, the students are as smart as the faculty. A smart student with self-confidence — and, as you know, I had a lot of self-confidence — knows what a good question is. And, a lot of the times, what I received from good questions was being put down, being put off. And I didn’t like that. At the same time, learning to deal with jerks is an excellent education in itself.

In the end, systems biology is a fancy name for what medicine is. When you’re doing any kind of experimental work in biology, it’s nice to connect it to what evolution had in mind, which is a workable organism. In theory, you can learn everything a medical student knows without attending med school, but it’s hard, right? You’d have to have incredible self-discipline. The first year or two of medical school, they basically attach a fire hose to your brain that fills you with useful facts. The basic science at Penn was great. I fell in love with physiology, which realized my dream as a 12-year-old, to know how the human body works.

In the end, med school was a great experience. I learned enough medicine to be the local “doctor” in my department at NIH. And knowing how to think about medicine, without keeping up to date with the state of knowledge, is useful when a someone you know has a disease that is not being diagnosed properly. And I would say, there are a couple of lives that I’ve saved, people in and around the lab, by providing a receptive ear to their medical issues.

Something else you may not be as cognizant of because you’re a med school faculty member. Just having an MD behind your name puts you in the MD club. And you can write to or call anybody in the world and get them to see a relative of yours, a friend, a friend of a friend — and that’s a huge advantage of going to medical school.

### NSG

Well, having been in a position where I needed to do that — it’s not everyone, but most people will eventually respond. And the other point I’d be interested in hearing your response to that I would make is that an MD is an incredibly broad education. And one of the things you get from doing patient-based work is you get a massive amount of information on one organism. And I don’t mean to demean people as patients by saying they’re an organism. The point being, that when you’re doing experimental mouse experiments or working with other lab animals you get a lot of information on each individual animal, but nothing like what you get on a patient. And so, it gives you an understanding of how you must integrate all these different types of information and how it can give you a deeper understanding of the complexities of biology — and the evolutionary aspect — has always been fascinating.

### JWY

For us, it was a bargain. We did the whole thing in 6 years. That’s the time it takes to get a PhD alone now.

### NSG

Most of the MD/PhDs at Case are taking 8 to 8 1/2 years. I think we were unusual in finishing it in 6, although maybe at that time, most people were finishing in 6.

### JWY

I finished it in 5 1/2; Walter was paying me as a postdoc for the last 6 months. I think that one of the highlights of my life was doing the MD/PhD at Penn. When students ask me today, “should I do it?” I answer that it’s different now. We could finish in 6 years and then some people didn’t even do a postdoc. If you did one, it was often only for a year or two. Also, most of us in the 70’s went straight from college to medical school. Students today often take a year or two off — say two years. If they do an MD / PhD, it’s at least 8 years; now up to 10 years. And then they often do a full residency. Now we’re up to 13 years. And a fellowship adds 2 years. And this is why the average age of getting the first R01 grant for a MD/PhD is 46! I was 30 when I got my first R01. If students had gone into investment banking, they’d be on their second retirement by then.

### NSG

Are there any particularly distinctive insights that have informed your research career and given you, from your perspective, a way of developing insights that other people don’t have?

### JWY

I give this “How to Succeed” talk, and I never say that a big part of success is being smart, but it is. A poignant story from early in my career. I went to a meeting in New York with other students in Walter’s lab. This might have been the first meeting I attended. I fell into the pattern I would have for the rest of my career. I attended the talks, but I wasn’t listening much, I wasn’t taking notes, and everyone could see that. On returning to the lab Walter, asked, “What did you learn at the meeting?” The other students didn’t really have much to say, and I homed in on the 2 or 3 most important things presented at the entire meeting. And the other students said “How could you know that? You weren’t even listening”. I just knew what was important. I still do.

A big chunk of being good scientist is having an innate ability to know what’s interesting and important. On top of that, to have creative ideas that lead to a doable experiment. There are smart people with great ideas that can’t be tested with the resources available. Having ideas that lead to experiments is a key part of success.

Something else I had a good feel for was not overthinking things. I had the right amount of “this isn’t a completely stupid idea.” In the end, I love doing experiments. And that was lucky, because I think the most important thing is to do experiments. To me, a hypothesis is an excuse to do an experiment. When you do an experiment a get some data it’s like walking through a magic door that enables you to think about everything much better.

The best people who’ve come from my lab have a similar philosophy. They do experiments — and they’re good at doing them, which is another important part of it; so, when you get a negative result, you don’t have to worry that you got it wrong. There are people who have great ideas leading to experiments they can do, but they’re so poor experimentally that when the experiment doesn’t work you don’t know whether to blame the experiment or the idea.

Something else that helped me succeed. As you know, Jack Bennink and I had a great partnership for a long, long time — 30 years. If you’re going to work with people, which is more important than ever, you need to share as much credit as possible. You need to receive enough credit to have a career. You need some fame in your own field, if nothing else, to get your papers accepted, and particularly to get money. But beyond that, fame is not useful. I would encourage people to give other people as much credit as possible. You can’t be completely selfless, either, because then you won’t have a career. You certainly can’t let people walk all over you and steal your ideas and get all the credit for it. But there’s a happy medium, like everything else in life.

The last thing is going to sound ridiculous. Don’t write grants! Once you’re in the grant writing system, you will end up doing a lot of science to please the people who give you the money instead of doing what you think is the most interesting and best science. As I’m literally wearing my NIH hat for this interview, I must acknowledge that I got lucky to wind up at NIH where, some of the listeners may not know, we don’t write grants. Intramural scientists must justify our research every 4 years, in 20 pages or less, to maintain our funding.

### NSG

As a concrete example of what you just said, we had a young faculty member at Case some years back who ended up leaving and going elsewhere. And she was doing excellent work. It was on a system that wasn’t considered as interesting, because at the time, HIV was rising in prominence, and it wasn’t HIV. She had developed a beautiful system with rabies virus looking at glycosylation changes on a viral glycoprotein where there was one glycan, and you could be confident that you knew what was happening. And instead, the study section pushed her to move to HIV, which was a nightmare for that kind of work, and maybe down the road that would have made sense once she had worked out a system where she had real control of all the various factors of interest. But it was, I thought, just insane to do that at that point. And, she wasn’t happy, but that’s the only way she could get funded.

### JWY

I wrote one grant when I was at Wistar as an assistant professor and the comments from the study section were ridiculous. I thought, “Who are these people? What do they know?” I was just starting my own lab, but it was the beginning of the antigen processing and presentation field. The grant was on mapping which viral proteins CD8+ T cells were recognizing in flu-infected cells, which in the end we did, of course. And some of the negative comments were that our proposal was too ambitious. What does that even mean? I think the worst thing you could say is that the proposal is too *un*ambitious. And, you know, grant writers aren’t stupid. I mean, they’ll figure out that they can only do so much. You propose a lot of ideas, because you’re not sure which ones are going to work.

## CAUSES OF IMMUNE RESPONSIVENESS

### NSG

In your view, does the immune system distinguish between self and non-self, employ some other dichotomous decision, or respond on the basis of a variety of variables?

### JWY

You and I have talked about this a lot. This is one of the things you’re most interested in, and I agree with you that it’s conditional and complicated.

### NSG

I would call it evolutionary pluralism. It doesn’t have any reason to care about just one way of doing whatever the goal is. If it works, evolution will exploit it.

### JWY

Yeah. It’s hard to put a number on it, but there are dozens of ways of knowing what’s an appropriate immune response and what isn’t. And then, of course, the immune system makes errors, right?

### NSG

It makes errors, and it’s also the case — I think you can make a reasonable argument anyway — that the immune response is not just about protection from invaders, it’s also about healing and repair, to some degree, and other physiological functions. Again, because evolution requires the complete integration of all physiological systems. And in fact, it can be difficult to even define what the boundaries of the immune system are.

### JWY

It is difficult. We had two papers on how the sympathetic nervous system influences flu immunity [7, 8]. And it’s arbitrary what we consider the immune system to be. A lot of the same molecules were discovered by multiple fields and given different names (TNF/cachechtin, IL1/pyrexin, etc.), which tells you that they’re important for more than one thing. And, you know, there’s probably not a single protein in all of nature that just does one thing.

### NSG

It’s one of my sort of pet notions that virtually any protein, if you look hard enough, will turn out to be pleiotropic.

### JWY

Even hemoglobin is doing other things, right? Likely, every protein is multitasking. And then, when you get to viruses, because of the limited genetic information — each viral protein has a long list of jobs.

### NSG

That’s a really interesting point, because viral genomes are so small, relative to cells, cellular genomes — even bacterial or mycoplasma genomes — they have to be incredibly dense in terms of functional information per unit of coding.

### JWY

Yes. We’re discovering now that there are all sorts of pervasive translation on the negative strand of negative strand viruses. We’ve not shown that any of these are functional, but sooner or later there’ll be a functional one, but translating proteins from the negative strand is happening all the time. It’s not surprising from a theoretical standpoint, because just what you said: There’s a tiny amount of information. For flu, it’s less than 15 kilobase per genome to outwit a human being with a 3 million kilobase genome.

## PRINCIPLES OF VIRAL IMMUNOLOGY

### NSG

Do you think it’s reasonable to say that there are any specific principles applicable to viral immunology that can be concisely formulated?

### JWY

That’s a hard one. The first principle, which is true of viruses and all pathogens, is that the immune system is remarkably good at limiting viral pathology. We know about the failures, but the successes are subclinical, right? Given how many viruses are out there, which is a very, very, very large number, the immune system is amazingly effective One of my favorite papers is by Curtis Suttle. He’s a marine virologist, and he had this *Nature* paper — and, you know I love numbers — and the number of bacteriophages in the ocean is such that if you line them up end to end, they extend10 million light years, 10X the size of the Milky Way [9, 10]!!

### NSG

And each one is what probably a few nanometers (actually tens to a few hundred).

### JWY

Don’t tell Fox News, but Suttle calculated that the largest source of CO_2_ in the atmosphere comes from bacteriophages lysing their host organisms. Viruses are everywhere, and so is the immune system, which is present in every dividing cell. It’s at least a standoff. And of course, evolution is complicated, and a virus that’s completely successful extincts itself. A virus has no interest in killing its host; it’s only interested in being transmitted to the next host. At the same time, the immune system does an amazing job of controlling viruses.

In fact, a lot of the viruses we worry about aren’t those that cause organ failure by killing cells. Many viral diseases are due to the immune system doing too much, not too little. You know, famously, in 1918, there was a “W” mortality curve; children died, frail elderly, with the upside down V in the W being people between the ages of 20 and 30. They weren’t dying because flu was killing all their lung cells. The healthy 18-30 died because their cytokine response was *too* enthusiastic. So, I think the immune system does a really good job.

Another generally applicable principle; immunodominance, which applies to B cells, T helper cells, T killer cells. In each case, the immune response to the relevant antigens is hierarchal [11, 12].

And that being said, I can’t pick a single virus where we have a detailed understanding of the protective immune mechanisms. We know which immune elements in mice we are required to prevent virus mortality. But we don’t know in mice or any animal model system how exactly the various elements of innate and adaptive immunity function to limit viral replication and minimize pathogenesis. Given the ethical constraints, this may be unknowable in a human being.

We did a lot of live imaging of cellular immunity in mice for about 15 years. It’s incredibly cool, but at the end you realize how difficult it is to understand what’s going on. Things are so crowded in organs, there are so many actors, everything’s moving around, and everything’s changing every second. It’s like trying to figure out music played by a billion instruments at once with the score changing every bar.

### NSG

Gives new meaning to the term dynamic scoring.

### JWY

Hah! It’s really complicated. But it’s not that you can’t discover things that are useful. Here’s a key difference between basic and translational science; in clinical science, you just want something that works. And then once you have it, you’ve succeeded. Basic science is easier in the sense that you’re going to discover something, biology is so complex it’s hard not to discover something. But then understanding it? “Understand” is an interesting word when applied to natural phenomena.

### NSG

This is why I would say that the concept of humility is always appropriate before nature.

### JWY

Absolutely. You’ll never know if you truly understand it, because you can’t know what you don’t know. Nature created us, and not the other way around.

### NSG

Is that just your way of bringing in Donald Rumsfeld’s taxonomy to epistemology?

### JWY

He wasn’t stupid.

### NSG

No, he wasn’t.

### JWY

In fact, Rumseld didn’t invent it, but I’m giving a talk soon and I use his “known knowns, etc. [13], which is a really useful way to think about things. And the unknown unknowns, might as well be infinite, for all we know. As a working scientist, that’s got to be your idea that there are always things that we don’t know. It’s even true for physics, where the smartest people are working on the simplest things. When you apply it to biology, where we are not quite as smart and everything is way more complicated, complete understanding is probably not possible.

### NSG

I just heard a podcast, MindScape with Sean Carroll, with a physicist who’s interested in the more conceptual aspects, and just trying to understand the concept of mass or gravity is not straightforward. Even though it’s an everyday word; we all think we know what it means.

### JWY

I know, I don’t know what it means. Once every couple of months, I try to understand what it means that the universe is expanding, and it literally blows my mind. As simple as the concept may be, I don’t understand it.

## ORIGINAL ANTIGENIC SIN AND THE ANTIBODY RESPONSE TO INFLUENZA A AND OTHER VIRUSES

### NSG

My next question was, what would you like immunologists to understand better about original antigenic sin?

### JWY

This is a quick and easy one. It’s memory. That’s all it is.

### NSG

The joke of it is that a lot of immunologists have forgotten memory.

### JWY

I taped a graph to my office door from a 1950 Frank Dixon paper that shows original antigenic sin in the rabbit antibody response to serum albumins. The history of antigenic sin goes back to at least 1909, when it was reported that patients infected with a given bacterial pathogen made a better antibody response to a prior related bacteria [14].

The immune system is not being stupid here. If there’s enough cross reaction to trigger a memory B cell, some of the antibodies, if not most/all, are going to be useful. Scott Hensley, a great postdoc in my lab who has gone on to a brilliant career published a wonderful paper in *PLoS Pathogens* about original antigenic sin. Scott showed the existence of “flu sin antibodies” that are protective against flu *in vivo* but not *in vitro*[15]. So, the sinful antibodies weren’t!

The immune system is not always perfect, there are all sorts of things that it gets wrong, like auto-immunity. But as a working hypothesis, the idea should be to figure out how the immune system gets it right. What are the evolutionary benefits of original antigenic sin? You taught me this Neil; you can only think about immunology, and everything else in biology, in the context of evolution.

### NSG

Well, I would say the selection pressure to be protected *in vitro* is limited.

### JWY

Very, very limited. Exactly right. You must figure that as a consistent feature of the immune system, original antigenic sin is probably useful. Let’s not call it something that makes us think its harmful, because maybe then we’ll do something dumb and try to eliminate something that has great utility.

### NSG

I saw another one of these news articles in, I think, *Drug Discovery News* or something, about imprinting. Which, suffice to say, as you pointed out, is just another word for memory.

### JWY

Thomas Francis was a brilliant scientist, and he came up with the term original antigenic sin [16] — he was a religious Catholic — that name is funny but dangerous because it makes you think about something in an unbalanced way.

### NSG

The problem is, I think some people are interpreting it in the wrong direction. I think all he meant by it was, in a very metaphorical sense, it was just an effect of a prior exposure, which is memory. That’s what memory is.

### JWY

That’s true. But “sin” is a bad word, made worse by its very cleverness.

### NSG

That’s right. But my point is that some people think that it was necessarily implying that there was some negative effect.

### JWY

Then don’t use sin. I’m sorry. I hate when people say, “it’s just semantics.” I mean, that’s how humans communicate.

### NSG

Exactly, and the point is, I don’t know what he intended, for sure because I didn’t know him, and I haven’t even been able to read his original article because I couldn’t get it.

### JWY

I’ll send it to you.

## THE RELATIONSHIP BETWEEN ANTIBODY AFFINITY AND VIRUS NEUTRALIZATION POTENCY

### NSG

Have you thought at all about the relationship between intrinsic affinity of an antibody and virus neutralization potency? What would you expect?

### JWY

It’s a simple expectation, as you know, for any given antigenic site (because it’s going to be different depending on the site,) there should be a linear relationship between affinity and neutralization in a given functional assay. But that has not been rigorously tested. And we’re going to do that experimentally, *in vitro* and *in vivo*.

### NSG

I think it’s important to do, because I suspect, although in certain cases, it may be a fairly simple relationship, I can easily see other factors intervening.

### JWY

I’m going to be extremely disappointed if it’s simply a law of mass action effect.

### NSG

I think it could be, in some cases, that is how it works. But I won’t be surprised if, in some viruses or for some antibodies, it’s more complex.

### JWY

We’re going to do it with a flu model system, we’ll take antibodies against different HA antigenic sites and test it. I think it’s an interesting question.

### NSG

It could depend on things like cross linking surface spikes, or monogamous bivalency could turn out to be important; it could be that there’s a limit past which the affinity doesn’t help much, etc. So, if you do find a simple relationship, it’ll probably be only over a limited range of affinities and so forth.

Why did you turn your interest to the cell biology of antigen processing years ago?

## CELL BIOLOGY: MRNA TRANSLATION, AND ANTIGEN PROCESSING

### JWY

I think my favorite course as an undergraduate was Cell Biology; I had a great professor, Fred Meins. This was the only course I took in college where I got the best grade in the class. Cell biology was something I was naturally good at. And then, as you know, I had the panel of anti-hem-agglutinin (HA) monoclonals that Walter had made [17].

### NSG

To influenza A virus, just for listeners.

### JWY

We made the first operational antigenic map and got lucky (although Walter probably knew what was going on) when the first structure of a viral protein was solved by Ian Wilson, Don Wiley, and John Skehel at that time [18]. Now, Ian’s justifiably famous and remarkably accomplished — he was a postdoc with Don Wiley. So that was his first great well-known contribution to science. Although he solved the structure of a different flu HA (H3 subtype), there was enormous structural conservation, not surprising now, but back then it was a surprise.

We sent the mAb escape mutants I had made to Andy Caton, a post-doc in George Brownlee’s lab, who sequenced them. When George and Andy located the mutations in the H3 HA structure (with help from the early structural biologists from Oxford) it turned out these antigenic sites were physically separated sites, which blew my mind. I was a postdoc in London when I first saw the paper. I’m an author on the paper [19] but I didn’t even see a draft.

Remember we had that giant magnetic board on a wall in Walter’s lab, and we were putting little magnets up as we were making the antigenic map? That magnetic map turned into an incredibly beautiful structure. I started to use these mapped antibodies with Thomas Bächi, Walter’s best friend from college who was a superb microscopist. He came one summer to Walter’s lab to do a mini sabbatical. And we really hit it off, becoming became lifelong best friends, even though he was 13 or 14 years older than me. Thomas was interested in the cell biology of viral entry, and so we started using the antibodies to study that [20]. It showed me these mAb can be incredible probes for studying protein conformational changes in cells. I learned more biochemistry and cell biology as a postdoc with David Lane at Imperial College in London, and then I taught myself more as an assistant professor at Wistar. I started doing pulse-chase experiments to look at HA biogenesis in combination with immunofluorescence. I taught myself to be a cell biologist looking at flu entry and HA biogenesis and export.

At the same time, I was working with Jack on which viral proteins were recognized by anti-flu CD8 T cells. Although our hypothesis was that T cell recognized native proteins on the infected cell surface I and Alain Townsend clearly showed that this was wrong and that T cells recognize short peptides from viral proteins.

Scooped, but not lethally. Townsend’s finding raised the question, how do the peptides get made? This was basically the start of the antigen processing field. There were a group of mostly young scientists interested in the cell biology of how antigen processing and presentation worked, and I was one of those people.

In essence, I had trained myself, inadvertently, to be in the perfect place to study MHC class I antigen processing with flu. Class I biogenesis, in some ways, is a lot harder than HA, because flu, like most viruses, when it synthesizes hemagglutinin, it’s approximately 10% of all the proteins that are being synthesized in an infected cell. So, pulse-chase experiments are easy due to the huge signal/noise. Although the class I synthetic rate is higher than the average cell protein, the signal is far lower than HA.

The biochemistry was harder. On the other hand, there was a super-sensitive functional assay for class I in the form of T cell lysis using the chromium release assay. In the early 90’s there was a lot more interest in antigen processing than any aspect of influenza virus. People got interested in flu again in the late 90s when bird flu panicked everyone, and then increasingly with each new potential pandemic threat. I had these really great papers on flu that no one was interested in, to be honest [21, 22].

I’m just interested in doing good science; I don’t care that much about the problem itself, as long as it’s fun and interesting, and so we switched the whole lab over to antigen processing and presentation and CD8+ T cell immunodominance. I didn’t start working again on flu until we inadvertently discovered a new flu protein looking for out-of-reading-frame class I peptides. And that was a good time to get back into flu. That was late 90s and as I said, bird flu had appeared, killing 6 people in Hong Kong. The world was starting to get frightened of flu and the scientific interest followed. And so, I had left flu for about 10 years and then gradually got back into it, to the point where the lab has been about 50/50 flu/antigen processing for 15 years.

## CLASS I MHC-RESTRICTED ANTIGEN PROCESSING

### NSG

What aspects of class I antigen processing do you think are insufficiently understood?

### JWY

Every aspect. Antigen processing is one of the fields where editors of high-profile journal have decided we know everything we need to know. There are still great papers published, but the world doesn’t read them or see them terribly much, because the top journal you can publish in is *Proceedings of the National Academy of Sciences*, which has antigen processing scientists on the editorial board who know how interesting and important the topic is.

While the world may believe that we’ve known everything about antigen processing for 15 years, I don’t. We certainly have the bare bones of the MHC class I pathway, and I think it’s likely that there aren’t any more dedicated gene products to be discovered. This is based on our recently published CRISPR screening paper looking for new dedicated class I processing genes that appeared back to back with a similar paper from Jacque Neefjes [23, 24]. Neither study found a new dedicated component of the pathway, suggesting that we know the major components.

One of the most mysterious aspects of antigen processing is critical for cancer immunosurveillance. The cellular immunopeptidome (the repertoire of peptides presented by class I molecules) is not simply based on the law of mass action. If it was, there’s an incredible number, something like 75% of the proteome, that is constituted by only 250 of the tens of thousands of proteins in the proteome. For T cells, it’s been reported that 25% of the proteome is constituted by only 12 proteins. Histones, ribosomes, translation factors, actin, tubulin, that’s mostly what the cell is synthesizing in terms of mass. And every protein made will be degraded. At steady state, it’s one-to-one, for every translation product synthesized one is degraded. If that was used for immunosurveillance, it couldn’t work. Because all that would be presented would be histones, ribosomes, etc. But that isn’t how it works. T cells can commonly recognize translation products where there’s no other way to detect the translation product.

Antigen processing breaks the law of mass action. I first realized this after showing that within 30 minutes of adding a virus to a cells, enough viral protein is made to enable CD8+ T cell recognition even though you can’t detect any viral proteins [25]. This has not been shown using mass spectrometry, where peptides are often detected in infected cells before their source proteins [26].

### NSG

In other words, the T cell, detecting fragments of translation products, is way more sensitive than our methods for detecting completed, native proteins.

### JWY

Right. And a lot of that is probably related to something that I named, and helped discover, although there were several labs that discovered basically the same thing. The name I came up with is DRiP, for defective ribosomal product [27]. The basic idea of DRiPs is that when you make a protein, you make more than the protein that’s going to be part of the proteome, more than what nature intended as a functional protein. While you’re making the protein, you’re making all sorts of mistakes that result in byproducts that are degraded rapidly generating peptides that are almost certainly selectively acquired by the peptide transporter in ways we don’t understand to enable rapid presentation and bypass the law of mass action.

### NSG

That’s really interesting, because what that basically says is that the T cells can detect not the native sequence necessarily, but an altered version of it. Which makes perfectly good sense evolutionarily. So, the T cell is taking advantage of reasonably repeatable (translation) errors that are just inevitable on a probabilistic basis.

### JWY

Yes, and there are now two “ultimate DRiP” peptides from flu that have been described — one discovered in my lab as an antigenic activity in HPLC fractions that took 20 years for the postdoc, Weisan Chen to define [28]. The other one was described this year by Ike Eisenlohr [29], who was the first postdoc in my lab and who has had a brilliant career in Philly at Jefferson and now Penn.

Ike’s peptide is incredibly cool because it’s presented by Qa-1, the equivalent of human HLA-E in mice. With MHC-E, there’s very little polymorphism. If this was common, Rolf and Peter would not be famous for discovering MHC restriction. In any event, Ike’s peptide and the one we found are very small gene products in the virus, 16 and 14 amino acid peptides. It’s not impossible that such a short peptide has some function, but as far as he and we can tell, the peptides don’t function to increase viral fitness. Knock it out, the virus doesn’t seem to care based on its normal replication.

These are the ultimate example of DRiPs, i.e., translation products that are useless to the virus. They likely arise because translation is not perfect, ribosomes don’t always start at the right place or stop at the right place. More generally, what are DRiPs? There are dozens of things that can go wrong in converting the information encoded in a nucleic acid into a fully functional protein. One interesting thing we’ve worked on is misincorporation of amino acids.

### NSG

Is this completely random?

### JWY

You hit the key point here, it can’t be completely random. It’s got to be beautifully random, right? Here’s a concept, you’re going to love Neil. There are enough amino acid substitutions in a protein that if a protein is more than a few hundred amino acids, the odds are very high it has one semi-random change, which means that proteins are statistical.

And you can easily imagine that nature is quite happy with this, because you’ve expanded your repertoire of protein functions enormously. If the mistakes you make are reproducible, then you can evolve a function.

### NSG

So, it’s really a sort of envelope of possibilities. And, essentially what you call the protein sequence is simply the average sequence.

### JWY

Yes, but by far the consensus sequence.

### NSG

It’s interesting, because it’s almost as if that’s a primary structural analogy to conformational fluctuation.

### JWY

It is. That is exactly right.

### NSG

I don’t think there are too many textbooks in biochemistry that would deal with that concept.

### JWY

This is a semi-field now, based on the idea that there are deliberate errors in amino acid incorporation. I think, in many ways, the single best paper from my lab, was a fantastic collaboration with Tao Pan at the University of Chicago [30]. We were looking for DRiPs that were related to antigen processing. We didn’t find that, but this is real serenDRiPity.

The story began with my reading a *Nature* paper I had had missed 7 years previously. — the first sequencing of entire human MHC, which helped kick off the human genome sequencing project. I’m looking at a gene map, and I’m interested in DRiPs, and there are tRNA genes everywhere in the MHC. tRNA in the MHC? It turns out, there are about 160 copies of tRNA genes in the human MHC. And more than 400 likely tRNA genes in the human genome that encode 264 different species. There are about 15 in each family with the same anticodon, but they’re all different. And evolution isn’t going to keep them for no reason. Why are they in the MHC? I had an idea, which turns out, as far as I know, to be completely wrong, that the translation machinery uses MHC tRNAs to deliberately make DRiPs.

Shortly thereafter, randomly, I had a phone call with Josh Plotkin, a smart bioinformatics guy interested in codon usage, who was working on HA. I told him my idea and he said, “Oh, you’ve got to talk to Tao Pan.” I find out who he is and call him. Tao had come up with a microarray-based method that could discriminate about 80 different tRNA families. We did this relatively simple experiment, labeled the cells with [^35^S]-methionine (Met) and asked where’s the Met — on which tRNAs? At that time textbooks said that the error rate of the enzyme — aminoacyl-tRNA synthetases—that attach amino acids to tRNAs which is the crucial step in maintaining decoding fidelity, is between 1 in 10,000-50,000. Okay, our first experiment, the error rate with resting cells is 1%. And then with flu infected cells, it’s 15%!

### NSG

Was this because of a different technique?

### JWY

Yes. Tao had this new technique that was much less work and more comprehensive.

### NSG

But how did those earlier numbers get…?

### JWY

It was all *in vitro*. But I don’t know why the numbers were so different

### NSG

But the point is, Pan had a different method.

### JWY

Yes. I’m not sure how they (i.e., the earlier investigators) got it so wrong. In any event, that was the start. This was a paper that took about 10 man-years of work. There are many co-authors from my lab whose experiments are not in the paper, because they didn’t work. We tried hard to establish that “Met-misacylation”, attaching Met to the wrong tRNA, had something to do with antigen processing, but failed.

In the end, we found something more important, the genetic code isn’t written in stone. And that it wasn’t only viruses that increased the mistake rate, any cellular stress did it, which we discovered by more serendipity.

Tao, before this, was exclusively working with yeast and bacteria and had never grown mammalian cells. So, we sent some cells to Chicago for them to use. Of course, they didn’t know how to do it at first. Coincidentally, we had this period where there was 15% baseline Met misacylation in both Tao’s and my lab. In his lab, it was because the graduate student didn’t know how to grow the mammalian cells. The cells were stressed because they were overgrowing them, they weren’t splitting them at the right time. And in my lab, we unknowingly had invented the most difficult assay for determining when a CO_2_ incubator is broke, which we detected by high Met misacylation!

### NSG

So, lab errors uncover translation errors.

### JWY

Yes! Well, we didn’t figure out the mechanism ourselves. We figured stress was involved but the breakthrough came when I told my friend Akiko Iwasaki, who was visiting NIH, about this. She started thinking about it, and made the key link. That’s a message here for young scientists: Talk to people. You never know what they know that you don’t.

Akiko emailed: “Jon, do you know Rod Levine at NIH?” I did, we had collaborated; but I hadn’t seen him in years. He is a world’s expert on oxidative damage to proteins. I didn’t know that. It turns out, Met has an ironic role in dealing with reactive oxygen species (ROS), which I also didn’t know, is induced stress. Any stressful event, including virus infection, turns on a huge ROS response. It turns out that Met is the most easily damaged amino acid. Evolution didn’t ignore that. There are enzymes that repair oxidized Met immediately. Rod had a theory, which we helped confirm, that Met is employed as what he called a “bodyguard” residue in active sites. If you need to protect a lysine or arginine or histidine from ROS damage you make sure a Met residue is nearby to neutralize ROS.

Akiko linked Rod’s theory to what we had discovered, and then Tao and others showed that it isn’t just mammalian cells that use Met misacylation when ROS is around, yeast, bacteria and archaea do it as well. So, we discovered a general way of bending the genetic code under conditions of stress.

This shows you how random it can be to make a great discovery. I was just reading a paper, because I was bored one afternoon, which led to a really good idea, which turned out to be completely wrong. Through a fortunate string of events, talking to the right person who linked me to Tao, talking to Akiko at exactly the right time, having the people in the lab who actually could do the experiments that were technically extremely difficult — it all led to this great discovery, which has nothing to do with what I thought it had to do with.

The whole problem with writing a grant is the idea that we do science to prove what we know, which is completely opposite of why we do science.

### NSG

Your friend could write the Tao of Translation.

### JWY

He has. While we were doing this, I was joking with him, “Tao, I’m going to make you famous.” In that field, I did. We still collaborate. He is a great scientist. The most fun you’ll ever have in science is when you find a collaborator whose skills are perfectly complementary to yours.

## CROSS-PRIMING

### NSG

What are the crucial aspects of cross-priming for the CD8+ T cell response?

### JWY

Cross-priming is crucial for cancer, for auto immunity. You need cross-priming, where you don’t have the danger signals for …

### NSG

Why don’t you first just define what you mean by cross-priming?

### JWY

To make a T cell response to viruses you need to activate naive T cells. There are, no doubt, exceptions, but naive T cells can “only” be properly activated by dendritic cells, as naïve T cells are much more difficult to activate than memory cells. Viruses infect dendritic cells, and I think my lab did the most work directly demonstrating that infected dendritic cells activate naïve T cells in lymph nodes. Chris Norbury, a post-doc in my lab, first used frozen sections of mouse lymph nodes [31], and then we extended the findings using intravital microscopy of live mice. This was the work of Heather Hickman [32–34], who came to my lab as a post-doc and stayed 15 years. She is a world class viral immunologist, who also is an outstanding intravital microscopist. She has her own lab now at NIH and is doing amazing things. Activation of naïve T cells by infected antigen presenting cells is termed direct priming.

But say you’re not a virus. For example, tumors can’t infect a dendritic cell. The idea is that there’s a process called cross priming, discovered by Mike Bevan, that antigen presenting cell (he didn’t know it at the time, but they’re dendritic cells, which were still to be discovered), can acquire a protein antigen and somehow introduce it into the class I pathway. That’s called cross-priming. What do we know about it? One, the predominant mechanism is based on a dendritic cell having phagocytosed, pinocytosed, or endocytosed (through absorption) a viral protein. We don’t know how an antigenic peptide is generated by the DC from the internalized protein. It could be generated in the endosomes, or by delivering the protein to the cytosol, which is what most people think. In either case, this is another event that breaks the law of mass action, because the amount of protein that’s internalized is miniscule compared to the amounts of competing cellular proteins being degraded by proteasomes

### NSG

Let me just break in and give a little bit of context here. So essentially, if I’m correct, what you’re saying is that the reason viral infection is important is because you have endogenous synthesis of viral products in the cell seeing infection as opposed to exogenous protein being taken into the cell that has been synthesized elsewhere that would normally be considered the class II antigen-presenting pathway. And yet, some of the peptides end up in the class I pathway.

### JWY

Exactly. And viruses do both.

### NSG

Actually, you may be interested, if you remember when I was in Peter’s lab — and Dave Schwartz was in Peter’s lab — we did an experiment, which showed that if we had UV-inactivated influenza virus or vaccinia virus, we could still prime for an *in vitro* secondary CD8+ T cell response. It was in *Infection and Immunity* in like 1984 [35].

### JWY

Oh, you know, I stiffed you, because we figured out how that works and didn’t refer to your paper. That was one of Jack and my early good papers. There was a guy named Yasuhiro Hosaka, who had spent a year or two at Wistar (Jack was his technician for a few months), who reported that heat inactivated flu sensitizes target cells for T cell lysis [36]. I figured out that heating works by inactivating neuraminidase, resulting in a huge increase in virion core protein delivery to the cytosol [37].

### NSG

Even though it’s not infectious, per se?

### JWY

To the extent you could prove it, we did that by showing first, that cells are still recognized when protein synthesis is blocked, and second that only proteins present on incoming virions are presented, and not a non-structural protein.

### NSG

It gets into the cell even though it can’t replicate.

### JWY

Absolutely. I first met Mike Bevan when he visited Penn in 1988 or so before publishing the cell biological mechanism for cross-priming. That was the first I heard of cross-priming. I told Mike what we had done with flu (the paper may have been in press) with that clue, Mike showed that ovalbumin can enter the class I pathway in the same way — not naturally as with flu entry, but artificially, by allowing cells to endocytose ovalbumin and then using a clever trick break open the endosomes releasing ovalbumin into the cytosol where it is processed and presented by class I to T cells [38].

### NSG

We might have done heat killed but I also believe we did UV-irradiated and antibody-virus complex.

### JWY

Antibody would help because it would increase internalization by professional antigen presenting cells. I did not know you guys had published that, which is cross presentation. But there are other mechanisms for cross-priming. There’s something I didn’t discover but I named: “cross dressing”, where a dendritic cell acquires MHC class I complexes by the process of trogocytosis And then it’s also likely that, as I alluded to before, that cancer cells, and other cells, are releasing exosomes all the time, and they have mRNA that can get into dendritic cells, and so, in that sense, the tumor peptide is presented like a viral peptide, and as much as it is being synthesized by the dendritic cell. And then Jacques Neefjes’ brilliant paper in *Nature* showed that dendritic cells can form gap junctions with other cells, and peptides can diffuse through the gap junction where they make the holes [39]. There are lots of pathways for cross presentation.

### NSG

A nice example for the pluralism of evolution.

### JWY

And then figuring out what’s doing what, in what system, is very hard, but cross priming is incredibly robust. Do you remember Barbara Knowles, a Professor at Wistar when we were students? She came to my lab for a summer on a mini-sabbatical, and we had a paper together [40]. She showed with Giorgio Trinchieri in 1976 that cross-priming with T antigen, the first defined viral T-cell target antigen, a weird one, because it can transform cells. So, you have these transformed cells that are highly immunogenic (this was the holy grail I futilely searched for in my undergraduate thesis). By the time Barbara came to my lab, Rolf Zinkernagel was claiming that cross-presentation is a weak, irrelevant phenomenon [41]. We submitted the paper which, as a reviewer said, was for one person, Rolf, which was kind of true. Because the paper only makes one point: cross presentation is robust. When Barbara first reported T antigen cross-priming in 1976, you couldn’t count T cells very well. By the time she came to my lab, it was possible to accurately quantitate the number of responding T cells by flow cytometry. Barbara, working with a really good postdoc, Weisan Chen, who found the crazy peptide discussed above, showed that when you immunize BALB/c mice i.p. with SV40-transformed B6 cells, at least 40% of activated peritoneal CD8+ T cells recognize defined T antigen peptides. The title of that paper was “Cross-priming is a robust phenomenon (Rolf) [40].

## IMMUNODOMINANCE

### NSG

Let’s move on to immunodominance.

### JWY

Jack and I spent about 25 years working on CD8+ T cell immunodominance. And I won’t say that we solved every problem, but we came up with the basic rules [42]. Number one is that some peptides are going to win. I guess that’s not surprising. But what’s surprising is the same peptides win every single time when you have a consistent genetic background. Or, even when you have the right class I allele in humans, most of the time, one peptide wins, but not quite as consistently. With CD8+ T cell responses in inbred mice, there’s rigid dominance hierarchy where there’s very little variation between mice and between experiments.

What’s it due to? Many things. What is the first protein expressed that generates the first DRiPs? How much of that protein is made? How easy is it for proteases to liberate the peptide? What is the affinity of the peptide for the class I molecule? How many T cells have a T-cell receptor (TCR) that could possibly respond to it? On top of all that, some peptide class I complexes stimulate T cells better, so they proliferate more.

People often say, “We know little about T cell immunodominance.” Ouch! Jack and I published dozens of papers and there are hundreds from other labs, and we don’t know anything about it? I know PIs must get grant money by pointing out what we don’t know, but still.

## SARS-COV-2 VACCINES

### NSG

Well, that’s why I’m giving you the chance to set the record straight. What’s your view on the implications of the rapid success in producing the vaccines for SARS-CoV-2, particularly the mRNA vaccines?

### JWY

One, Warp Speed was a great thing; it saved a lot of lives.

Two, a bigger story: COVID vaccines were a huge triumph for immunology and virology. And people were like, “Well, why do we spend all this money on NIH?” The number of lives saved by effective COVID vaccination [43] not to mention therapeutic agents for HIV, pays many times over for all the money spent on all the biomedical research ever funded by NIH. We couldn’t have done that without all the money we plowed into basic research. No way.

### NSG

If you built a tree of all the findings that led into that, it would probably stretch into dozens of areas: cell biology, virology, immunology, biochemistry.

### JWY

It’s a triumph, but we we’re lucky that coronavirus is easy to target.

### NSG

This is what I think is crucial that some people are getting wrong. Every virus is not going to be as straightforward.

### JWY

Do you remember how bad the Wistar mouse colony was? There was Sendai Virus; mouse hepatitis virus (MHV). At one point, we couldn’t do any experiments because MHV, which is a mouse coronavirus, was infecting every mouse and you can’t measure a CD8+ response to other viruses in a mouse with MHV infection. Jack made his own MHV vaccine by simply inactivating a MHV stock with beta-propiolactone. We immunized mice when they arrived from the vendor, and we had no MHV. You can make the MHV vaccine in an hour in your own lab. Human coronaviruses are easy vaccine targets. I’m not taking away from the mRNA vaccine, which worked better than an inactivated coronavirus vaccine does, but still, inactivated coronavirus vaccines will probably work well enough to end an epidemic if everyone would be immunized. Humanity was lucky that SARS-CoV-2 was such an easy target.

### NSG

And it’s not even just that, it’s that SARS-1 is incredibly similar to SARS-CoV-2. It was the same basic spike structure.

They already knew what antigen to focus on. They already knew what mutation to make to stabilize the structure.

### JWY

Almost any recombinant virus would work, e.g. the J&J adenovirus vaccine. Contrary to common belief that the mRNA vaccine was miraculously effective, other vaccines worked, but not quite as well.

### NSG

You’re right. I get your point. I think the mRNA approach has a lot of advantages in terms of scaling up.

But the point is, with a new virus family or even a new virus, you wouldn’t necessarily know what antigen to focus on, you wouldn’t necessarily know how safe it was.

### JWY

Well, if you go for the virus receptor protein, most of the time it’s going to work. And then a lot of inactivated vaccines just work. All you must know is how to grow the virus. Polio vaccine works. It’s not perfect, in fact, the inactivated virion vaccine is better than the replicative vaccine for polio, but way more expensive. That’s the barrier. The beauty of the live polio vaccine is that each dose is a few pennies.

### NSG

So, that suggests going back to the work of Jonas Salk, who produced the inactivated vaccine, because the live vaccine still causes real polio in a tiny fraction, but it’s still enough people that it’s not worth doing.

### JWY

No. And you inadvertently reintroduce strains that would have been extinct otherwise, right? That happened. Not because of stupidity, it is just the way things go. You can’t know everything. But Coronaviruses are easy targets, and that was not a surprise from animal work. Most viruses are easy targets, to be honest. I mean, you’re not necessarily going to get a durable vaccine to a respiratory virus. At this point, there is no durable vaccination for viruses that cause mucosal-based disease [44]. But you’ll get a vaccine that ends an epidemic and that saves lives.

### NSG

Well, that’s a beautiful segue into the next question, which is do you expect us to have an effective vaccine for HIV in the next 10 years?

## VACCINE DEVELOPMENT FOR HIV

### JWY

Maybe the cytomegalovirus (CMV) vaccine — the vaccine that Lou Picker, Klaus Früh, and a great group of scientists are working on in Oregon [45].

This vaccine works by inducing a crazy noncanonical T cell response. They’re CD8+ cells recognizing MHC-E (a non-classical human MHC class I molecule), presenting peptides that don’t appear to bind MHC-E with high enough affinity to be presented. None of it makes sense, except that 50% of the rhesus monkeys are either cured or prevented from getting infected with SIV (the monkey equivalent of HIV) in the first place. They’re doing human trials now, initially to test immunogenicity.

Beyond that I don’t know of other promising HIV vaccines. Antibodies are hard for a lot of reasons. The virus itself is challenging, because completely sterilizing immunity is necessary. That means not a single cell is infected, since it only takes one virion to be integrated into the genome, and it’s over. HIV reactivates when host T cells divide, and the virus population will expand slowly. Over 10 to 20 years, it will kill you. The problem with HIV is you have to neutralize every single virion. With an antibody, that’s hard. T cells in theory can kill every infected cell. In practice, they can’t because of the latency of HIV. And there is an idea that a T cell vaccine for people who are already infected is going to work, which is how any T cell vaccine would have to work. There’s no evidence that’s going to work. It could work in naive people, I guess, because it’s at least theoretically possible. But in somebody who already has HIV, who already is making a huge fraction of their T cells against HIV, making more is almost certainly not going to be curative.

## DEVELOPMENT OF A UNIVERSAL INFLUENZA VIRUS VACCINE

### NSG

What are your expectations for the success in the next 10 years for the development of a universal flu vaccine?

### JWY

Very low. T cells are not going to work, because of the reason I just said, people already have a lot of flu-specific T cells. And there’s something about mucosal immunity, we don’t understand. It doesn’t last very long. And there’s no real evidence that IgAs are this magical, antiviral antibody that a lot of people are saying now. The most common immunodeficiency in Europeans is in IgA — there are some people who have very little IgA, and they don’t do much worse with respiratory infections. Further, while there are conserved regions (like the HA stem) that can be targeted, it is likely that the virus will find a way to escape.

In the long run, while it might be possible to eradicate influenza B viruses because they only infect humans [46], influenza A viruses have an enormous animal reservoir and are impossible to eradicate.

### NSG

And apparently expanding.

### JWY

Isn’t that crazy? I just read, I think in *The New York Times* that the reason that cows are an issue is that cells in the utters have the right glycans for binding flu, enabling flu to infect those cells, at least that’s the simple idea. But cows were not known to be a target for flu or a vector for flu. And, not to mention sea elephants and sea lions, which have been dying from flu. It was known that seals can get infected, but this this is a scary virus. It is killing lots of animals.

In the end, knowledge is good, right? That’s the motto of the fictional Faber College in Animal House. And we’re not going to be less likely to make a flu vaccine by learning more about the basic principles of immunology, and particularly mucosal immunology. But at this point, we don’t know enough to have any confidence that a future vaccine is going to work better than what we have. And I’ve heard informally that the mRNA vaccines may not be better for flu. Yet. Maybe there are tweaks. And one of the advantages of an mRNA vaccine is you can add all sorts of antigens, if you like, you can add all sorts of cytokines, if you like. That’s one of the great advantages: You can mix a whole bunch of mRNAs and very easily manipulate the immune response; really tailor it.

## GRADUATE AND POST-GRADUATE RESEARCH TRAINING

### NSG

It is an incredibly versatile method for making vaccines. Next question: how do you believe graduate and postdoctoral research training can be improved?

### JWY

First, I think we made a huge mistake by confusing our training pool with our labor pool. And the fact that we have this grant-based system that we’ll get to in a few minutes, basically means you’re contracting for work, which means the people doing the work (graduate students and post-docs) are the sub-contractors. This is a major conflict of interest in that the work that the professor must get done to maintain a career and a livelihood — and particularly since universities don’t pay salaries anymore on their own — conflicts with the needs of the trainees, which only partly align with the needs of the grant. We expanded the number of graduate students to get the grant-mandated work done, which is also a mistake. If the US government wants grant-generated knowledge, it should put enough money in the system to hire professionals to do the work.

Some of those professionals are postdocs, who we need to pay way more money. We’re finally getting closer to fair salaries for their expertise. We’ve created a system where you go to graduate school for six or eight years, and then earn less than if you would have entered the labor market straight out of college. That never made any sense whatsoever. How did this system arise? I think that it springs from the MD mindset. MDs have had by far the largest impact on the NIH system (every NIH director has been an MD). MDs underpay and overwork interns and residents and have always done so. This system was grafted on to the PhD training system. Completely stupid, right? And one of the things that irritated me in medical school — going back: the idea that students must stay up all night when taking care of patients to learn what it takes to be a dedicated physician. You don’t need to do that. This tradition is an excuse that allows hospitals to save money by hiring one doctor instead of three to take care of patients. And they grafted this philosophy to postdocs, which is why postdocs have been underpaid forever. Not a huge fan of unions, in general, although I understand you absolutely must have them because employers will take advantage, but I am happy that post-docs are now joining unions. As you know, Neil, I’ve been advocating for increased postdoc salaries for a long time [47].

Jeff Frelinger, the Chair of UNC Microbiology/Immunology, at the time was in my lab doing a mini-sabbatical in the late 90’s. He’s the one who hooked me up the with AAI policy committee, where he and I eventually had an impact on NIH post-doc salaries at the national level. I was the voice; he was the force behind it. And Jeff told that me that when he went back to UNC, had discussions with his department faculty about raising postdoc salaries, while his department members were uniformly liberal politically, when it came to their own post-docs, they acted like McDonald’s owners who would pay their post-docs the minimum wage, if possible. The post-doc system is unethical. It’s finally changing, because so few PhD students now are doing postdocs.

### NSG

They go straight to industry.

### JWY

I don’t blame them. I am all for post-docs unionizing, because they fought to improve working conditions without unionizing, and got nowhere for the past 30 years. If an employer is going to be unreasonable, then people must unionize. I am completely in favor of that. I think graduate students unionizing is a big, big mistake. Because then they’re saying they want to be treated like employees, which they are not. They are trainees. And we push them to this but treatment as employees is wrong. But still, you will not be successful scientists if you’re thinking of yourself as an employee. It won’t work.

### NSG

Maybe it’s not impossible, but they’re not totally compatible mindsets.

### JWY

You’ve got to be in love with science; you must be engrossed by it.

### NSG

I have a colleague in my department, Vincent Monnier, who has a kind of rule of thumb. He asks trainees — students or postdocs — do you think about your work afterhours? Because if you’re not interested enough to continue thinking about it, when you’re not physically in the lab, then this probably isn’t the right direction for you.

### JWY

You won’t be able to make discoveries, because you must be obsessed, no matter how smart you are. You need two things to make important discoveries: you need the high-powered intellect, and you need the obsession. Either alone won’t do it.

Another problem: we’ve never rationally thought about how a biomedical research lab should be structured. Scientists insist on evidence-based decisions. We need evidence-based design of the research lab. What is the best lab structure for productivity and for how happy people are? Happiness is rarely considered in how we should redesign biomedical research. One of the silver linings of COVID is that it accelerated acceptance of the idea that careers need to be tempered with happiness. The younger generations aren’t going to accept the career status quo. They want to be happy. And here’s the thing with science: You’re obsessed with it, because you love it, which makes you happy. You’re not unhappy because you’re not super rich. We need to pay young scientists a living wage, and we can debate what that should be. But if science is what you love doing, you’re going to be a happy person doing science, if the sacrifice is not too steep.

### NSG

Yeah, as long as you’re making progress, it’s very rewarding.

### JWY

It’s unbelievable. I have zero regrets about my career. I mean, none. I’m lucky. I found what I love. Completely lucky.

## SCIENTIFIC COMMUNICATION

### NSG

Do you have any thoughts on how to improve the quality of scientific communication?

### JWY

I think we’ve come a long, long way. If you remember when we were students, the talks were terrible. PowerPoint, which is a bit dangerous because it tends to oversimplify things, made talks 1000% better. There’s much more emphasis now on communication. Everybody gives a great talk today. It used to be that virtually no one did. We had an antigen processing meeting 5 years ago, and one of the guys said, “we’ll do lightning sessions; everyone gets 3 minutes; Jon, I want you to chair it because you’re going to keep everyone on time”. I replied, “this is never going to work; it’s going to be a disaster”. I was completely wrong. I didn’t have to keep anyone on time except the old people, who gave terrible talks; I had to stop them after 3 minutes after they had gotten 1% in. There were 2 older scientists and about 28 younger scientists. Twenty-eight people gave talks in under 3 minutes that were fantastic. I think the younger scientists are being educated now from day one, on how to give a good talk. And they do. And we also give them feedback. When you and I were students, there was no feedback. There was no help. Slides were a disaster.

### NSG

It was painful to make slides.

### JWY

You had to sit down with an artist. It was expensive. They would always make a mistake. It would take a month or two to get the mistakes worked out.

### NSG

I remember, even as a postdoc, getting these little letters you to rub onto your poster one at a time.

### JWY

Well, my in undergraduate thesis; it’s my terrible hand-drawn graphs. I think communication skills have come a long way. On YouTube, for example, there are people even without PhDs, who are marvelous communicators, who talk about things I know about and get almost everything right. I think scientific communication is not the major factor in public ignorance of science.

## ANONYMOUS VERSUS SIGNED MANUSCRIPT REVIEWS AND PREPRINT PUBLICATION PLATFORMS

### NSG

Do you have any strong feelings about anonymous versus signed reviews?

### JWY

Very strong feelings. I really believe that reviewers must be anonymous to prevent favor banks and all sorts of bias in the review, because reviewers fear retribution. It’s fine if you love the paper. But if you don’t like it, it’s opening a can of worms. There is a very good reason why reviews were anonymous. And this idea that everything is better served being out in the open is completely wrong — with every phase of life. There are times when things need to be open. But there are lots of times where you don’t want to open up everything. It is going to make life worse and not better, if that’s our goal.

### NSG

I mean, would you acknowledge that in the anonymous review process that people can scuttle other people’s work and get away with it?

### JWY

Nothing is perfect. I have a solution to the anonymous reviewer issue: get rid of reviews! bioRxiv and medRxiv? I love them. If I had to choose between the archives and the current review system, I would get rid of peer review for basic research papers, since if they are wrong, there won’t be an immediate negative medical impact. I think the baggage, work, and agony that goes with the review process is not worth it at this point. It’s a huge amount of labor that we’re donating our time to for free, which is insane. What other profession donates, whatever it is — 10 hours a week — to maintain the reviewing process. Do lawyers do that? Does anybody do that? If you’re a non-professional journal editor, you are unpaid or paid way below your market value. It’s insane.

I believe that the review process adds very little value to papers from good labs, where all of the problems have been discussed extensively in lab meetings and one-on-one meetings. In my experience, most papers I review and almost every paper I’ve published, there’s not much improvement during the review process.

### NSG

Sometimes there’s improvement, but as often there’s the reverse. They make it worse. They force you to say things that aren’t quite right. Or they force you to do experiments that make it harder to follow the thread of the paper.

### JWY

True. One thing I found that works as part of the process. There are experiments I want a post-doc to do, and they just don’t want to. And I figure that a reviewer will make them do it, and that usually works. But again, the experiment is not something critical or we wouldn’t have submitted the paper.

Here’s an irony: if we switch to a non-peer-reviewed process, it’s actually going to make it more difficult for lesser-known scientists to get attention to their work. Because, with the imprimatur of the journal’s prestige, the only way to distinguish a good paper from a bad paper is to read the paper and actually think about it.

The advantage of the current system is that a young scientist, or scientists from a department that no one knows, have a shot at the prestigious journals where people are going to read their work. Branding, ironically, is going to get worse than it is now if we switch to a non-peer-reviewed system. What we have now is not working. And the profit margins of for-profit publishing companies are obscene.

### NSG

And they’ve continued to consolidate like other areas of the marketplace. There’s just a handful (of companies) that control most of the premier journals.

### JWY

It’s like 3-cup monte. You send your paper to *Nature*, where it’s free to publish, and they’re like, “Oh, we can’t publish it because it’s under cup 3, but we can publish it for $10,000 in *Nature Ish* under cup 2.

### NSG

Right. Just some page charges, that’s all.

### JWY

It’s a racket, right? We can do better than this. Scientists are too passive. We must organize and decide we’re not going to put up with this anymore. NIH now is mandating that by 2026 papers must be free to read upon publication if NIH is funding the research. So, there are going to be some massive changes in the scientific publishing industry. And now is the time to try to reform the system for the better.

## BIOMEDICAL RESEARCH FUNDING

### NSG

So, the second-to-last question is: If you had the authority and means to do so, what changes would you make in biomedical research funding?

### JWY

I have three proposals: First, split NIH funding into 2 firewalled pools — the National Institutes of Basic Biomedical Research and the National Institutes of Clinical Research. The problem is that the clinic keeps taking away from basic research. The way to solve that is to separate the money. And obviously, there’ll be some tweeners. But we need some way of making sure that everything doesn’t go to translational research, because that’s the way it’s going now. The second is to use the NIH Intramural model, where you hire a good scientist and you fund them and make sure they’re productive, as opposed to a grant where you fund a project. The current system works because there are so many good people and there’s so much money, but its inefficient and highly stressful for everyone. The intramural system has been in place in NIH since 1950. It is a better system.

### NSG

I wrote a letter to the editor, I think of *FASEB Journal*, years ago with Dave Kaplan, where we proposed, basically, that Howard Hughes funds people not a project and that it would be better if everybody was under that kind of a system. But in the meantime, instead of giving more NIH money to Howard Hughes people who don’t need it, they should have had Howard Hughes pay the NIH for the peer review.

### JWY

That would have been a fairer system. My third proposal is going to be more controversial.

The science that you and I and everyone does, it’s great. And important for humanity. But at this point, in the developed world, we’re looking at marginal gains in human health. The major problem with humanity right now is behavioral. People exhibit behaviors that reduce their health, happiness, and lifespan. And the problem with disinformation is enormous.

What I’m proposing is to create a well-funded National Institute of Psychology, not to take money from NIH, but have a separate pool of money so we can figure out how to get people to do what we know is beneficial for their health. For example, many won’t take vaccines, which have the highest therapeutic index of any medical intervention ever invented by many orders of magnitude. This is insanity. And you and I could spend the rest of our lives, or young you and me, our whole careers, making a better vaccine, but what good is that if someone won’t take it?

### NSG

Along with that, you could say that a good fraction of all the medical problems in this country are created by the profit motive in the food industry and cigarettes. Basically, people make vast fortunes by addicting people to goods or services.

### JWY

That’s also part of it, Neil. But the disinformation is part of that as well.

### NSG

Absolutely. Well, they’re making money from disinformation.

### JWY

We need to understand more about how to get people to make the right choice. So that’s my 3-pronged approach.

## ADVICE FOR YOUNG PEOPLE INTERESTED IN SCIENTIFIC CAREERS

### NSG

Last, what advice would you want to give to young people who are potentially interested in pursuing scientific careers, particularly in biomedical research?

### JWY

Six points.

1. Only do it if you love it. If you think you’re working too hard, you don’t love it.
2. In your choosing a lab, particularly as a graduate student, pick the mentor more than the project.
3. Focus on learning the scientific method; experiments, experiments, experiments. This is how you’re going to learn how to be a scientist.
4. No one will read your dissertation. I know that. But put all your pride into it. It’s an incredibly valuable exercise in making yourself confront what you’ve done in a historical context and teaching yourself how to write. I think the time spent in writing a dissertation pays off massively in the rest of your career. Not just that, some of the best reviews of topics I’ve ever read are the opening chapters in dissertations. I’ve encouraged students to submit these chapters as review articles, which I know has been done at least several times. If you write an outstanding thesis, you could have 1 or 2 review articles that will establish your independent publishing career as well.
5. When you’re looking for a postdoc, do your homework. If you go to postdoc in a bad lab, it’s your fault. There are labs where no one is happy, where 10% of the people wind up getting a job that they want, yet people still go to these labs. They’re famous labs. But do your homework. Find out what happened to the people who went to those labs. Find out what your career prospects are.

### NSG

This goes back to your earlier point about talking to people.

### JWY

Yeah. Talk to people who have left the lab. When people are interested in coming to my lab, I give them a list of every person who has been in my lab and their email addresses, so they can talk to them about what it’s really like. Because sometimes you go to visit the lab, and the lab members are so intimidated by the PI, they won’t give you an honest opinion.

Point 6. Read my book. I wrote an eBook on how to succeed in science, it’s 99¢ on Amazon (they don’t allow free eBooks), or it’s free if you email me. I’ll send you a link to a file that you can read on an eBook reader or a Kindle. I’m not saying that the book has all the answers for young scientists; it doesn’t. But it will at least get readers thinking about the important factors in having a happy career. And again, we can’t define success simply by how many papers you published, or how important they are. A critical part of success is how happy you are doing it. In some ways that’s the main thing. When you run a lab, the lab runs a lot better and is a lot more creative when people are happy. It brings out the best in everyone’s behavior and everyone’s thinking when they’re happy.

### NSG

That’s a good point. It’s hard to be creative if you’re miserable.

### JWY

But not impossible! There are all sorts of biographies. Some of the articles I enjoy reading the most are the histories that people write about their careers that appear in the Annual Review series. I get a kick out of knowing the life stories of people. Funny, what I liked most about medicine was taking patient histories. I like that about scientists as well — knowing the hurdles they faced and how they overcame them and who was helpful and who wasn’t, and the tips they have for other people.

### NSG

The other aspect you get out of that is that you see how hard it is to hit on the right idea. How many ways you can go wrong.

### JWY

Going wrong is often going right.

### NSG

That’s true, too. But, in other words, how complex biological phenomena turn out to be and how easy it is to end up doing something other than what you expected.

### JWY

I think that’s the goal of every experiment. In a really successful experiment, you’re thinking about something you had never thought of because you found something new. That’s the whole point of doing basic research — to find something new.

### NSG

Okay, well, thank you, Jon. That was a fantastic survey of interesting topics in immunology. At least I hope people find it fascinating. And appreciate all of your time. We’ve spent quite a bit of time here today.

### JWY

That’s about normal for our phone calls though Neil.

### NSG

Just another phone call.

### JWY

Pretty much. Not that different. Thanks to you, much more organized, that’s all.

### NSG

Thank you very much.

## Figures and Tables

**Figure:**
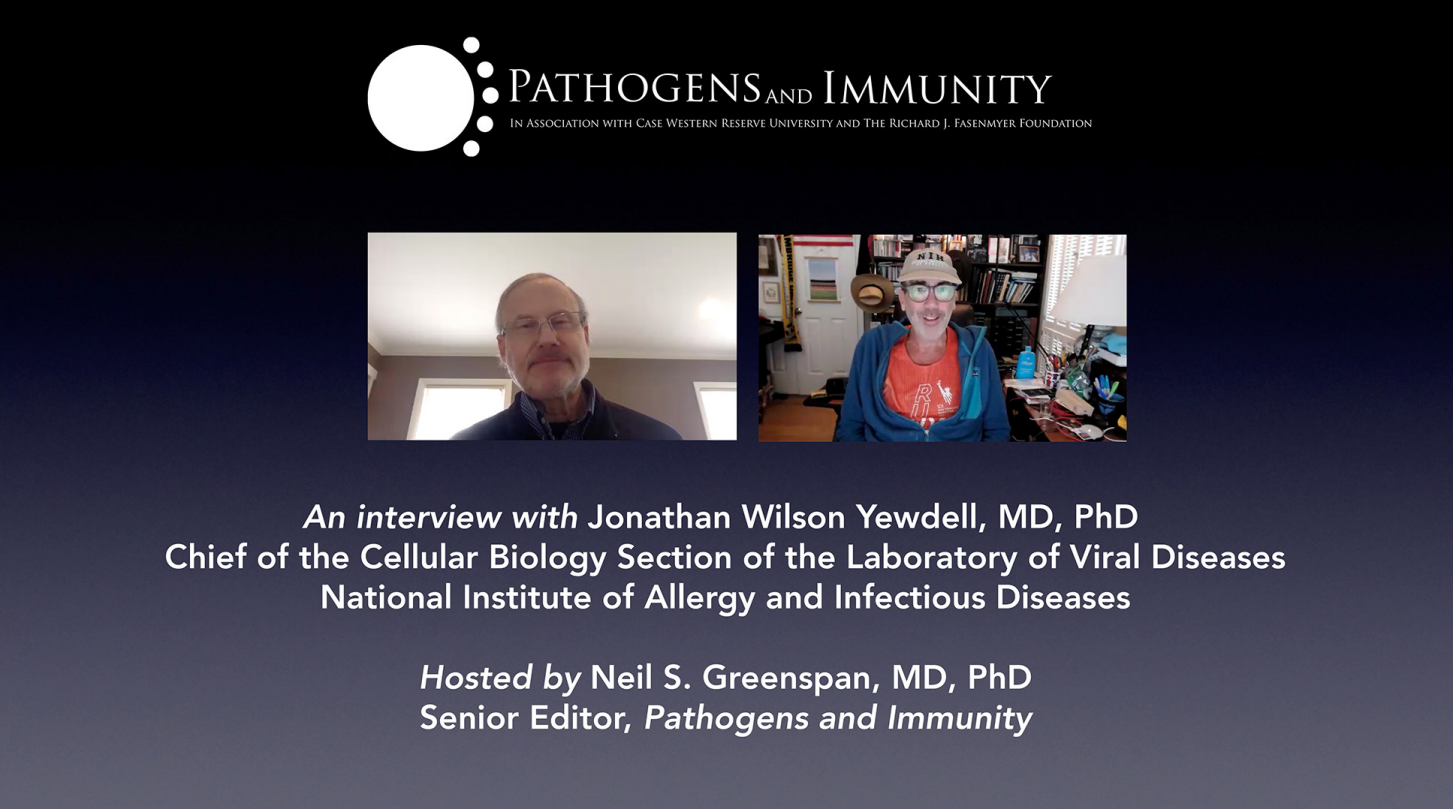


## SUPPLEMENTARY DATA

Supplementary materials are available at the *Pathogens and Immunity* website. Supplementary data may be provided by the authors to benefit the reader. Supplementary data are not copyedited and are the sole responsibility of the authors. Questions or comments related to supplementary materials should be addressed to the corresponding author.
